# Role of antenatal plasma cytomegalovirus DNA levels on pregnancy outcome and HIV-1 vertical transmission among mothers in the University of Zimbabwe birth cohort study (UZBCS)

**DOI:** 10.1186/s12985-021-01494-3

**Published:** 2021-01-29

**Authors:** Kerina Duri, Simbarashe Chimhuya, Exnevia Gomo, Privilege Tendai Munjoma, Panashe Chandiwana, Louis Marie Yindom, Kudakwashe Mhandire, Asaph Ziruma, Sekesai Mtapuri-Zinyowera, Lovemore Ronald Mazengera, Benjamin Misselwitz, Felicity Zvanyadza Gumbo, Sebastian Jordi, Sarah Rowland-Jones, for (UZBCS) The U Z Birth Cohort Study Team

**Affiliations:** 1 Department of Immunology, University of Zimbabwe College of Health Science (UZ–CHS), P.O. Box A178, Avondale, Harare, Zimbabwe; 2 Department of Paediatrics and Child Care, UZ-CHS, Harare, Zimbabwe; 3 Department of Medical Laboratory Sciences, UZ-CHS, Harare, Zimbabwe; 4 Nuffield Department of Medicine, University of Oxford, Oxford, UK; 5 Department of Chemical Pathology, UZ-CHS, Harare, Zimbabwe; 6 Department of Obstetrics and Gynaecology, UZ-CHS, Harare, Zimbabwe; 7 National Microbiology Reference Laboratory, Harare Central Hospital, Harare, Zimbabwe; 8 Clinic for Visceral Surgery and Medicine, Inselspital Bern and Bern University, Bern, Switzerland; 9 Department of Gastroenterology and Hepatology, University Hospital Zurich and Zurich University, Zurich, Switzerland

**Keywords:** Antenatal CMV-DNAemia, HIV-1-RNA load, Birth outcomes, Infant health, HIV-1 vertical transmission

## Abstract

**Introduction:**

Despite being a leading infectious cause of childhood disability globally, testing for cytomegalovirus (CMV) infections in pregnancy is generally not done in Sub-Sahara Africa (SSA), where breastfeeding practice is almost universal. Whilst CMV and human immunodeficiency virus (HIV) are both endemic in SSA, the relationship between antenatal plasma CMV-DNA, HIV-1-RNA levels and HIV-1-mother to child transmission (MTCT) including pregnancy outcomes remains poorly described.

**Methods:**

Pregnant women at least 20 weeks’ gestational age at enrolment were recruited from relatively poor high-density suburbs in Harare, Zimbabwe. Mother-infant dyads were followed up until 6 months postpartum. In a case–control study design, we tested antenatal plasma CMV-DNA levels in all 11 HIV-1 transmitting mothers, as well as randomly selected HIV-infected but non-transmitting mothers and HIV-uninfected controls.

CMV-DNA was detected and quantified using polymerase chain reaction (PCR) technique. Antenatal plasma HIV-1-RNA load was quantified by reverse transcriptase PCR. Infants’ HIV-1 infection was detected using qualitative proviral DNA-PCR. Predictive value of antenatal plasma CMV-DNAemia (CMV-DNA of > 50 copies/mL) for HIV-1-MTCT was analyzed in univariate and multivariate regression analyses. Associations of CMV-DNAemia with HIV-1-RNA levels and pregnancy outcomes were also explored.

**Results:**

CMV-DNAemia data were available for 11 HIV-1 transmitting mothers, 120 HIV-infected but non-transmitting controls and 46 HIV-uninfected mothers. In a multivariate logistic regression model, we found a significant association between CMV-DNAemia of > 50 copies/mL and HIV-1 vertical transmission (*p* = 0.035). There was no difference in frequencies of detectable CMV-DNAemia between HIV-infected and -uninfected pregnant women (*p* = 0.841). However, CMV-DNA levels were higher in immunosuppressed HIV-infected pregnant women, CD4 < 200 cells/µL (*p* = 0.018). Non-significant associations of more preterm births (< 37 weeks, *p* = 0.063), and generally lower birth weights (< 2500 g, *p* = 0.450) were observed in infants born of HIV-infected mothers with CMV-DNAemia. Furthermore, in a multivariate analysis of HIV-infected but non-transmitting mothers, CMV-DNAemia of > 50 copies/mL correlated significantly with antenatal plasma HIV-1-RNA load (*p* = 0.002).

**Conclusion:**

Antenatal plasma CMV-DNA of > 50 copies/mL may be an independent risk factor for HIV-1-MTCT and higher plasma HIV-1-RNA load, raising the possibility that controlling antenatal CMV-DNAemia might improve infant health outcomes. Further studies with larger sample sizes are warranted to confirm our findings.

## Key point

Antenatal plasma CMV-DNA of > 50 copies/mL may be an independent risk factor for HIV-1 vertical transmission in resource limited settings.

## Introduction

Cytomegalovirus (CMV) is a common infection in humans. The typical disease course is subclinical in at least 90% of primary or non-primary infections in immunocompetent hosts. However, following infection, CMV persists indefinitely in human hosts [1]. In developing countries, there is near-universal CMV sero-prevalence from infancy [2]. On the other hand in developed countries CMV sero-prevalence increases with age, with infection rates of 36%, 50% and 91% among 6–11, 30- and 80-year-olds, respectively [3].

Even in immunocompetent hosts the significance of CMV infection is increasingly implicated in comorbidities, since CMV seropositive individuals have systemic immune-dysregulation associated with increased risks of cardiovascular diseases and pneumonia [4–6]. CMV infection is even more important in immunocompromised hosts such as individuals with human immunodeficiency virus (HIV) infection and graft recipients, in whom CMV can cause severe disease such as pneumonitis [7] and meningitis [8]. It remains an important co-infection in HIV-infected adults even among those on successful combination antiretroviral therapy (cART) with good viral control, and has been associated with increased morbidity and mortality, including the development of non-AIDS-defining illnesses (9;10). CMV reactivation or disease may be under-reported in high CMV/HIV sero-prevalence settings where access to CMV screening and treatment are limited due to poverty, compounded by the unavailability of such services in public health care facilities.

CMV reactivation often occurs during pregnancy, more frequently in HIV-infected women including those on cART [1, 2]. In our clinical setting, infection is nearly universal with 99.6% of pregnant women showing chronic (non-primary) CMV infection [11]. However, preconception CMV immunity provides limited protection as intrauterine transmission can occur in women who are sero-immune prior to pregnancy [12]. CMV has been shown to replicate at the uterine-placental interface, potentially impairing vascular remodelling thereby occluding blood flow causing local hypoxia and other adverse foetal outcomes [13].

CMV is the leading infectious cause of congenital abnormalities and permanent disabilities. CMV-MTCT rate is 2% in developed countries with up to 15% of the infected new-borns being symptomatic, presenting with microcephaly and intrauterine growth restriction [14]. Furthermore, up to 15% of the initially asymptomatic infants progress to develop long term morbidities such as non-genetic sensorineural hearing loss, severe motor developmental and visual impairment as well as varying degrees of adverse neurodevelopmental outcomes, including cerebral palsy, seizures and intellectual disability [15]. CMV also remains a risk factor for childhood acute lymphocytic leukaemia [16]. Despite all these health problems and/or disabilities, there is poor awareness of congenital CMV (cCMV) among women of reproductive age in both developed and developing countries [17–19]. Furthermore, the exact pathogenesis remains largely unknown but could be due to a complex interplay between, placental, foetal, maternal and viral factors [20].

The role of maternal CMV replication on adverse pregnancy outcomes and infant health remains poorly understood in SSA with near-universal adult CMV infection and high HIV-1 sero-prevalence. Furthermore, in the same setting breastfeeding is the norm regardless of maternal HIV status. It is plausible that maternal CMV reinfection and/or reactivation may be contributing to the relatively higher HIV-1 vertical transmission rates and other adverse pregnancy outcomes common in this setting, but this has not been adequately investigated. In this pilot study we investigated the association between antenatal plasma CMV-DNA levels and HIV-1 vertical transmission events, plasma HIV-1-RNA load and pregnancy outcomes in a subgroup of pregnant women ≥ 20 weeks gestational age participating in the University of Zimbabwe Birth Cohort Study (UZBCS).

## Methods

### Design of University of Zimbabwe birth cohort study (UZBCS)

The UZBCS aims to investigate the role of maternal comorbidities, including co-infections with persistent viruses such as HIV, CMV, hepatitis B virus, and other infectious or non-communicable diseases including maternal nutritional status on pregnancy outcomes, infant mortality, development, immunity and health. By design, approximately 50% of the expecting women the UZBCS are HIV-infected. Briefly, at enrolment all women answered a structured questionnaire aiming at a comprehensive clinical, socio-demographic, environmental and household characterization of the research participants. Further, socio-economic information comprising employment status, family monthly income, money set aside for food were recorded including general household food security. Date for cART commencement and compliance were recorded in HIV-infected women. According to Zimbabwean national guidelines, all HIV-infected women should be on lifelong cART (Option B +), but in practice pregnant women often do not receive a timely HIV diagnosis. The current standard of care for all Zimbabwean HIV-infected pregnant women to prevent HIV MTCT consists of (non)–nucleoside reverse transcriptase inhibitors; TENOLAM-E (Tenofovir, Lamivudine and Efavirenz. Mothers breastfeed as long as they wish to. However, exclusive breastfeeding is encouraged during the first six months of life.

All HIV exposed infants are commenced on Cotrimoxazole prophylaxis until they stop breastfeeding or they test HIV-PCR positive, whichever occurs first. Upon detection of HIV-1 infection, infants are immediately commenced on cART, usually comprising the protease inhibitors Lopinavir/ritonavir and nucleoside reverse transcriptase inhibitors, Zidovudine and Lamivudine.

The UZBCS recruited 600 HIV-infected and 600 HIV-uninfected pregnant women at least 20 weeks of gestation from February 2016 until June 2019. Mother-infant dyads will be followed for 2 years, with an expected study completion date in June 2021.

### Selection of participants at enrolment into UZBCS

Potential participants for the cohort were identified during routine antenatal care visits at any one of the four out of a total of twelve City of Harare Polyclinics, situated in the South-western high-density areas of Harare namely, Kuwadzana, Rujeko, Budiriro and Glenview. Residents of these communities are of relatively poor socio-economic status. Follow-up visits of the mother-infant dyads were performed at delivery, within 10 days of life and 6, 10, 14 and 24 weeks postpartum. Date and mode of delivery, birth weight and length, feeding practices and information on the general health and development of infants were also collected.

### Selection of participants for CMV analysis from UZBCS participants

Data and bio-samples were available from the first 527 mothers recruited consecutively from February 2016 and August 2017, of which 280 were HIV-infected. Due to financial  limitations, CMV-DNAemia measurements were only possible in a subgroup of pregnant women. We first selected all mothers who transmitted HIV to their infants by 6 months of age (n = 11) as MTCT cases. A total of 120 HIV-infected but non-transmitting mothers were randomly selected as controls by a computer Pseudo Random Number generated algorithm. Three of these women gave birth to live twins. Depending on respective analyses’ outcomes we referred to this group as 120 HIV-infected but non-transmitting mothers or 123 infant-mother dyads. An additional 46 HIV-uninfected controls and their infants were also randomly selected using the same procedure.

### Blood collection and analysis

Ten millilitres of maternal venous blood was collected at enrolment. Maternal HIV diagnosis was done using qualitative rapid immunochromatographic assays, SD Bioline HIV-1/2 3.0 (Standard Diagnostics Inc., Kyonggi-do, South Korea) and Abbott’s Determine*®* HIV*-*1/2*.* Western Blot was the tie breaker for any indeterminate test results.

Full blood counts were determined from whole ethylenediaminetetraacetic acid (EDTA) blood samples using a Mindray© Haematology 3-part differential, 16 parameters BC3600 Analyser (Shenzhen, China). For diagnosis of anaemia in pregnancy, the World Health Organisation (WHO) definition was used (haemoglobin < 11.0 g/dL) [21].

Absolute CD4^+^ T-lymphocyte counts in EDTA blood samples were enumerated within a maximum of 6 h after sample acquisition for all HIV-infected mothers using a Partec Cyflow counter (Cyflow, Partec, Munster, Germany).

### Maternal CMV/HIV detection and quantification

For analysis of HIV-RNA and CMV-DNA loads, blood was centrifuged for 5 min at 3000 rpm and the plasma was aliquoted in appropriately labelled cryo-vials and stored at − 80 °C until testing.

Total CMV nucleic acids were extracted from 200 µL plasma using the QIAamp MinElute Virus Spin Kit (Qiagen, Hilden, Germany). Then, 60 µL of total viral nucleic acids including CMV-DNA were eluted and immediately stored at − 80 °C for subsequent testing. CMV-DNAemia detection and quantification was performed by quantitative PCR (qPCR) using the RealStar CMV PCR kit v1.0 (Altona Diagnostics, Hamburg, Germany). CMV-DNA was quantified using the QuantStudio 3 Real-Time PCR System (Applied Biosystems, CA). The detection limit was 1 copy per mL.

For HIV, viral nucleic acids were extracted from 1 mL maternal baseline plasma and HIV-1-RNA quantified using an automated TaqMan Roche Amplicor 1.5 Monitor Test (Cobas AmpliPrep/Cobas TaqMan, Roche Diagnostics, Branchburg NJ), according to the manufacturer’s instructions. The detection limit was 20 copies per mL.

### Assessment of adverse birth outcomes

Adverse birth outcomes included preterm birth (< 37 weeks of gestation), low Apgar scores at 5 min (< 7) and low birth weight (LBW, < 2500 g), weighed within the first hour of life, before significant postnatal weight loss had occurred. Head circumference and birth lengths were also measured in centimetres. Z-score transformation of infant birth weight and birth length followed normal distribution of WHO reference data of 2006. Transformation was performed using the R package “zscorer”.

### Early infant HIV diagnosis

Venous blood (EDTA) or heel prick blood spots were collected and preserved on 903 protein saver card (number 105311018) at delivery, 10 days and 6, 10, 14 and 24 weeks. Every infant blood sample of each study visit was processed and stored as dried blood spots and plasma at − 20 and − 80 °C, respectively for further evaluation, including HIV and CMV assessments. Infants’ HIV infection was detected using a qualitative 1.5 Roche Amplicor HIV-1 proviral DNA PCR kit (Roche Diagnostics Incorporation, Branchburg, New Jersey).

### Data management and statistical analysis

Data were entered and managed using Research Electronic Data Capture (REDCap v 8.0, © 2020); http://www.redcap.uzchs.ac.zw/redcap/. Quality assurance on the accuracy of data entry included independent double entries and verification in cases of discrepancies.

Parameters from groups of HIV-infected/-uninfected and/or CMV-DNA positive (viremic) and negative (aviremic) women were compared using the Kruskal Wallis test, Mann–Whitney U test, Chi-squared test and Fisher exact test where appropriate. For all serology positive test results, undetectable levels of HIV-1-RNA load or CMV-DNA were assigned the value zero. For multivariate linear and logistic regression analyses, the following predictors: CMV-DNAemia of > 50 copies per mL, HIV-1-RNA load, years since HIV diagnosis, ongoing cART use duration and maternal age, all at the time of enrolment were tested.

For infant outcomes, all infants (including twins) were included, and for each infant the respective maternal parameters were used (resulting in usage of the same maternal parameters for both twins). For maternal outcomes (e.g. prediction of maternal HIV-1-RNA load) the mothers with twin deliveries were counted once.

Automated parameter elimination for optimization of the Akaike information criterion using the “glmulti” R package was done. Due to limited statistical power, we did not consider interaction between predictors. All calculations were performed in R Studio 1.2.5001.

## Results

### Study participants

From 527 pregnant women recruited between March 2016 and August 2017, 280 (53%) were HIV-infected and 247 (47%) were HIV-uninfected.

**Fig. 1:**
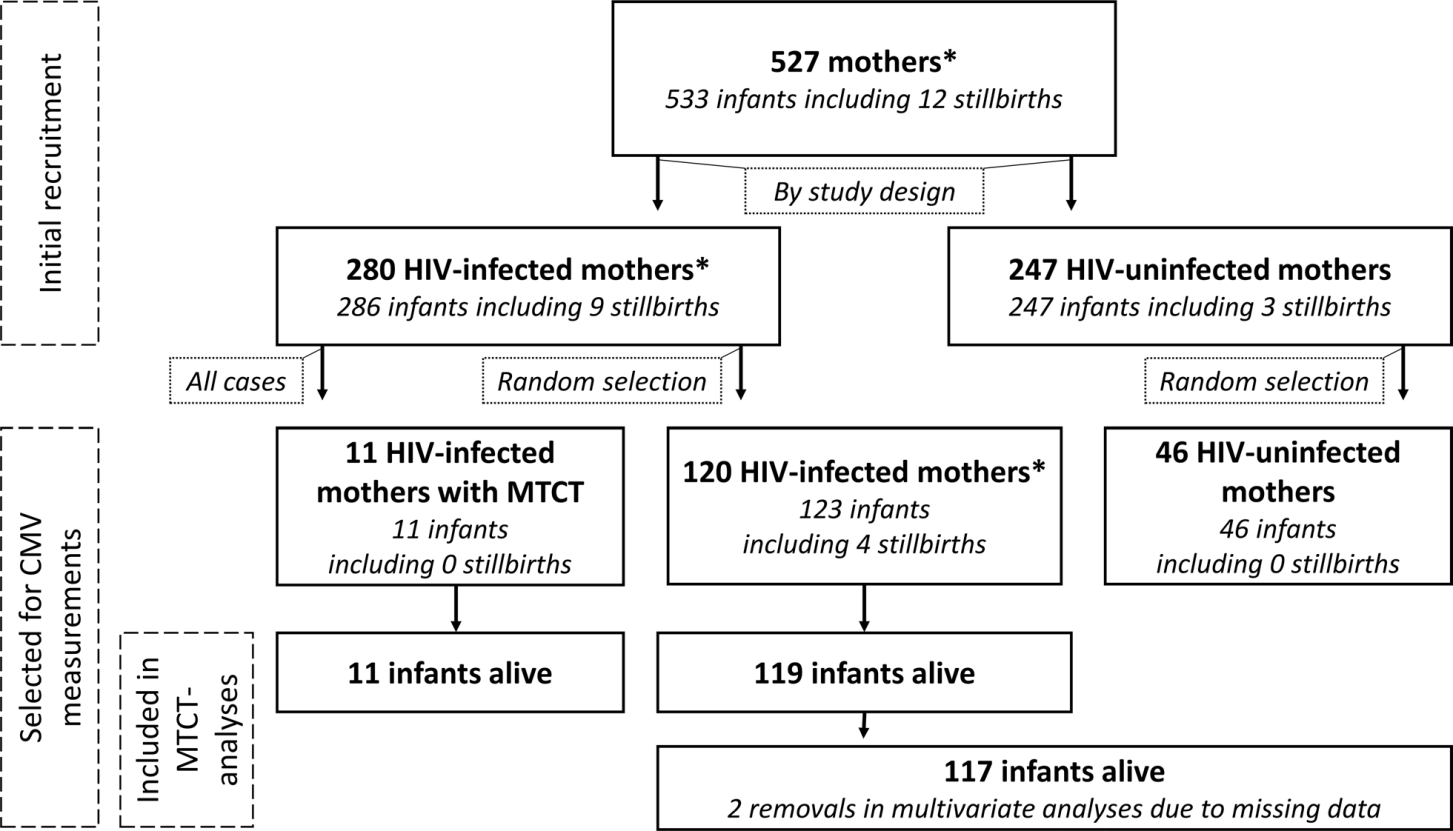
Flow chart of mothers’ subgroups. Flow chart illustrating mothers’ subgroups for different analyses. * Differences between numbers of mothers and numbers of infants are due to twin pregnancies

For the antenatal plasma CMV-DNA analysis, we selected all 11 HIV-infected mothers with subsequent HIV-MTCT as cases (HIV transmitters) and randomly selected 120 HIV-infected but non-transmitting mothers and their 123 infants (3 twin pregnancies) as controls, as well as 46 HIV-uninfected mothers and their 46 infants (no twin pregnancies). A total of twelve stillbirths were observed, of which 9 were HIV exposed but were not tested for in utero HIV infection, Fig. 1.

No relevant differences were observed between the randomly selected mothers included in the CMV-DNA analysis and the rest from the UZBCS population (Additional file 2: Tables 1, 2).

Baseline characteristics stratified by HIV status of all the pregnant women included in the CMV-DNA analysis (N = 177) are presented in Table 1. HIV-infected pregnant women were significantly older (*p* = 0.031) but showed similar anthropometric measures and socio-demographic characteristics with regards to family income, household size and number of infants < 5 years old living in the household. As expected, HIV-infected pregnant women with CMV viremia had lower haemoglobin levels (*p* = 0.02), Additional file 2 : Table 3).

**Table 1:** HIV-infected and uninfected study participants

Variables Median (1st quartile-3rd quartile); min–max		Stratifications; HIV status and CMV-DNAemia; N = 177			
Maternal HIV status		HIV-uninfected mothers, N = 46		HIV-infected mothers including vertical transmitters, N = 131	
CMV-DNAemia		CMV aviremic, N = 36	CMV viremic, N = 10	CMV aviremic, N = 99	CMV viremic, N = 32
CMV viremic (detectable)	Yes	N/A	10(21.7%)	N/A	32(24.4%)
	No	36 (78.3%)	N/A	99(75.6%)	N/A
CMV-DNA (copies/mL)		N/A	178 (126–344); 82–30,279	N/A	345 (207–500); 30–3169
Maternal age (years)		24.4 (21.7–29.8); 18.1–41.7	26.3 (22.5–34.1); 17.6–40.1	29.5 (25.2–34.2); 18.4-.43.7	28.3 (23.3–32.6); 18.3–41.7*
Mid upper arm circumference (cm)		28.5 [26–30]; 20–42	27 (25.3–28.8); 23–35.5	28 [26–30]; 21–39	27.3 [25–30]; 22–35
HIV-RNA (copies per mL)		N/A	N/A	195 (20–10784.5); 0–257539**	1665 (20–81, 150); 0–614706
CD4 count cells/µL		N/A	N/A	404.5 (277–517); 55–966*	341 (122–470); 26–1153
Pregnancy outcomes (N = 180 infants, including 3 sets of twins)		CMV aviremic mother, N = 36	CMV viremic mother, N = 10	CMV aviremic mother, N = 101	CMV viremic mother, N = 33
Delivery < 37 weeks gestational age	Yes	7 (19.4%)	1 (10%)	22 (21.8%)	12 (36.4%)
	No	29 (80.6%)	9 (90%)	79 (78.2%)	21 (63.6%)
Birth weight (g)		3075 (2737.5–3325); 1800–3885	3000 (2925–3037.5); 2600–3900	2910 (2600–3250); 1300–4230	2800 (2500–3300); 1000–3600
Birth weight z score (WHO Growth Reference 2016)		− 0.6 (− 1.3-(-)0.0); − 3.8–1.1	− 0.7 (− 0.9-(-)0.7); − 1.7–1.1	− 0.9 (− 1.7-(-)0.2); − 5.5–1.5	− 1.2 (1.9-(-)0.1); − 6.8–0.5
Birth length (cm)		49(48–51); 42–58	51 (49.3–52); 47–56	49.(48–51*); 36–55***	49 (48–50); 27–56 *
Birth length z score (WHO Growth Reference 2016)		− 0.1 (− 0.7–0.6); − 4.2–4.8	0.8 (− 0.0–1.5); − 1.5–3.7	− 0.1 (1.0–0.6); − 7.3–2.7 ***	− 0.5 (− 1.0–0.5); − 12.1–3.7 *
Vertical transmission by 6 months of age	Yes	N/A	N/A	5 (5.2%) ††††	6 (18.2%)*
	No	N/A	N/A	92 (94.8%) ††††	27 (81.8%)

Pregnant women characteristics, stratified by HIV status and CMV-DNAemia, 4 groups. Data are expressed as N (%) or median (IQR); min–max unless stated otherwise. For calculation of vertical transmissions only infants that survived the first 6 months of life were included; exclusions due to death are marked with one “†” for each deceased infant. P-values to compare groups were calculated using the Kruskal Wallis test, Mann–Whitney U test or Fisher’s exact test where appropriate. Missing data are indicated in the following manner: number of * = number of missing data points. Maternal age (years), Vertical transmission by 6 months of age both p values = 0.03, otherwise all the other p values are > 0.1

BMI: body mass index, CMV: cytomegalovirus, HIV: human immunodeficiency virus, WHO: world health organization, N/A: not applicable

Of the 131 of HIV-infected pregnant women, regardless of the vertical transmission status, 28 (21%) had their first diagnosis of HIV done at enrolment into our study, and hence they were cART naïve. 103 were already on cART at enrolment, and of these, 27 (26%) were on cART for < 30 days, [median (1st quartile-3rd quartile); min–max]; [11 [2–24]; 1–29] and 76 pregnant women were on cART for > 30 days, [799 (141–1677); 32–4327], with self-reported cART adherence rates of 100% and 87.5%, respectively. HIV-RNA load of ≥ 1000 copies/mL were observed in 54/129 (42%) of the pregnant women (2 cases of missing data). As expected, cART use was strongly associated with lower HIV-RNA load, but no further trends were detected (Additional file 2: Table 4).

HIV exposed still born infants not tested for in utero infection were excluded from HIV-MTCT analysis, observed in 11 infants (4.0%). The timings of HIV infection of these infants were 1 (9%) and 3 (27%), in utero* and* intrapartum, born of the cART naïve mothers and < 30 days duration of cART use groups, respectively. The remaining infants 7 (64%) were infected through breastfeeding and were all born of mothers with > 30 days duration of cART usage. Of these 7 postpartum transmitting mothers, 2 had HIV-1-RNA loads of < 20 copies/mL.

### CMV viremic and CMV non-viremic pregnant women

Plasma CMV-DNA was detected at comparable frequencies in HIV-uninfected and -infected pregnant women (HIV-uninfected: 10/46, 21.7%; HIV-infected: 32/131, 24.4%; *p* = 0.841), although a non-significant association of higher CMV-DNA levels with HIV-infection was observed (Table 1).

In a multivariate analysis, neither HIV status nor maternal age nor socio-demographic parameters such as number of children under five years in the household were significantly associated with CMV-DNA load (Additional file 2: Table 5). CMV-DNA load of > 1000 copies/mL were observed in 6 out of 177 women (3.4%), whilst higher load of > 10,000 copies/mL was detected in only one HIV-uninfected woman. CMV-DNA levels were significantly higher in immunosuppressed pregnant women with CD4 < 200 cell per µL (*p* = 0.018, Mann–Whitney U test).

### Association of CMV DNA levels with HIV vertical transmission

**Fig. 2:**
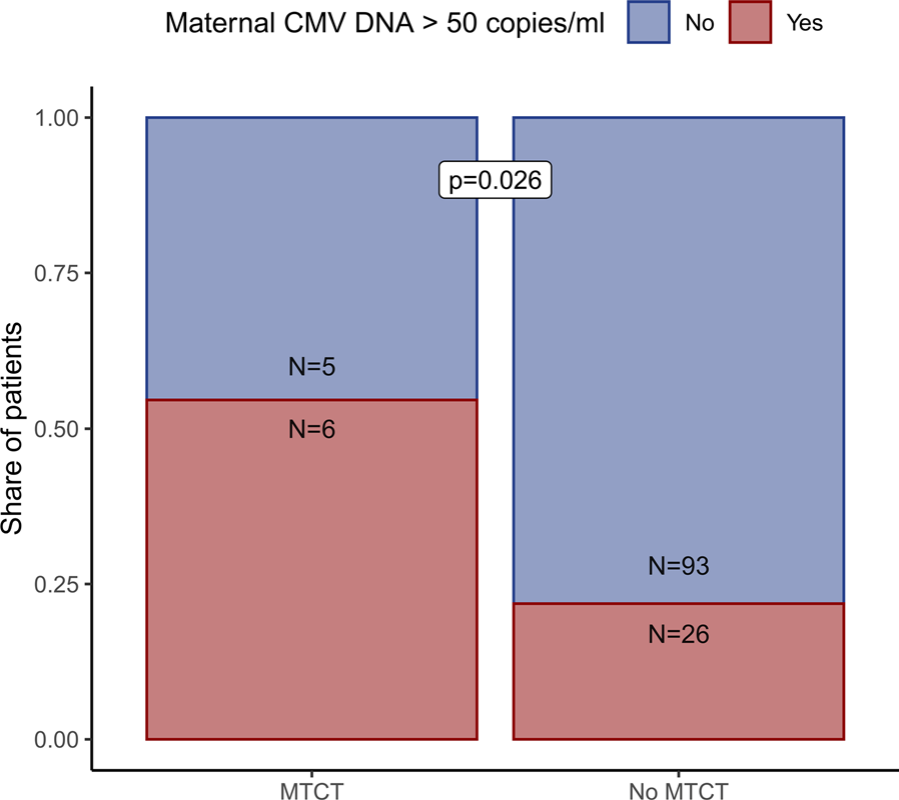
Association of HIV-MTCT and maternal CMV-DNA levels. Illustration depicting the relative shares of mothers with CMV-DNA of > 50 copies/mL stratified for HIV-MTCT status. P-value was calculated using Fischer’s exact test

In our pilot study, 130 infants born of HIV-infected pregnant women were examined for HIV-MTCT (Fig. 1). 11 with vertical HIV transmission events were included as cases. In comparison with the control sample of 119 infants without MTCT, CMV-DNAemia was associated with HIV-MTCT, since MTCT was observed in 6/32 (19%) CMV-DNA viremic mothers compared to only 5/98 (5.0%) of CMV-aviraemic mothers (*p* = 0.026, Fig. 2).

This association was confirmed in a multivariate analysis. A non-significant association of higher HIV-MTCT with mothers having CMV viraemia of ≥ 50 copies/mL and MTCT independent of maternal plasma HIV-1-RNA load was observed before parameter elimination (OR 3.62, CI 0.81–16.29, *p* = 0.085, Table 2). However, after parameter elimination, the association of CMV viremia ≥ 50 copies/mL at enrolment with HIV-MTCT was significant (OR 4.02, CI 1.09–15.30, *p* = 0.035, Table 2).

**Table 2:** Predictors of HIV-MTCT

	Dependent variable: HIV transmission (yes/no)	
	Without variable elimination	With variable elimination
*Logistic regression results—odds ratios for vertical HIV transmission risk*		
Maternal CMV-DNA of > 50 copies/mL (yes/no)	3.62 (CI 0.81–16.29) *	4.02 (CI 1.09–15.30) **
Maternal HIV-RNA load (per 1000 copies/mL)	1.05 (CI 0.99–1.14)	
Years with HIV diagnosis	1.09 (CI 0.93–1.26)	1.12 (CI 0.97–1.30) *
On cART (yes/no)	1.08 (CI 0.96–1.22)	
Maternal age (years)	3.81 (CI 0.39–167.23)	

Logistic regression showing the association of different variables with MTCT in HIV-infected mothers with live infants. Results are presented as odds ratios; p-values are indicated as follows: *p < 0.1, **p < 0.05, ***p < 0.01. 128 infants were analysed (excluded: 2 due to missing data, 4 stillbirths)

CI: 95% confidence interval, CMV: cytomegalovirus, cART: combination anti-retroviral treatment, HIV: human immunodeficiency virus

### Association of CMV with plasma HIV-1-RNA load

**Fig. 3:**
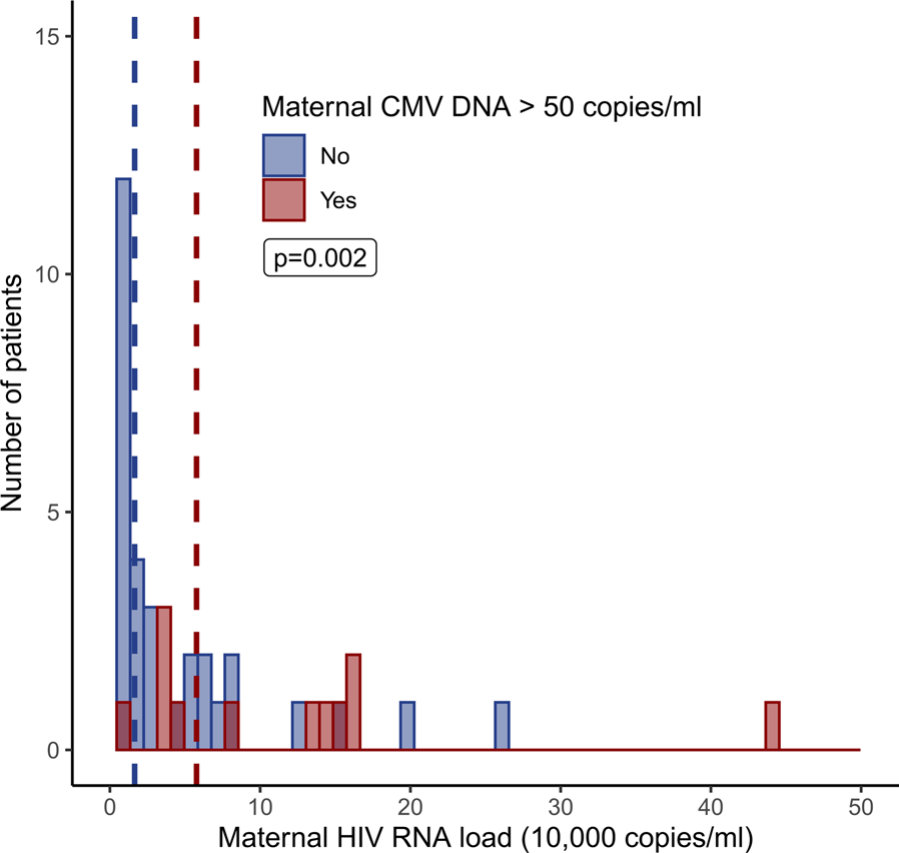
HIV-1-RNA loads stratified for CMV-DNA. Bar plot of mothers’ HIV-1-RNA loads stratified for maternal CMV-DNA of > 50 copies/mL. Dashed lines indicate groups’ means

Associations of different parameters with plasma HIV-1-RNA load were examined in HIV-infected but non-transmitting mothers. In a multivariate linear regression analysis, CMV-DNAemia of ≥ 50 copies/mL and also CMV-DNA load (copies/mL) as continuous variable were significantly associated with higher HIV-1-RNA load (both *p* = 0.002) while as expected cART usage correlated with lower HIV-1-RNA load (Table 3, Fig. 3, Additional file 1: Fig. 1). These parameters remained significant after automated parameter elimination.

**Table 3:** Predictors of maternal HIV-1-RNA load

	Dependent variable: maternal HIV-RNA load (10,000 copies/mL)	
	Without variable elimination	With variable elimination
*Linear regression results—linear coefficients for antenatal HIV-RNA load*		
Maternal CMV-DNA > 50 copies/mL (yes/no)	4.15 (CI 1.57–6.74) ***	4.07 (CI 1.53–6.65) ***
Years with HIV diagnosis	− 0.10 (CI − 0.47–0.27)	
On cART (yes/no)	− 3.32 (CI − 6.05-(-)0.60) **	− 3.58 (CI − 6.04-(-)1.11)***
Maternal age (years)	< 0.01 (CI − 0.19–0.19)	

Linear regression showing the association of different variables with maternal HIV-1-RNA load in HIV-infected but non-transmitting mothers. Results are presented as linear coefficients per 10.000 HIV copies/mL; p-values are indicated as follows: *p < 0.1, **p < 0.05, ***p < 0.01. 118 mothers were analysed (excluded: 2 due to missing data, 4 stillbirths and 11 MTCT cases)

CI: 95% confidence interval, CMV: cytomegalovirus, cART: combination anti-retroviral treatment, HIV: human immunodeficiency virus

### Association of CMV levels with delivery before 37 weeks

We tested whether CMV levels were a predictor for preterm births before 37 weeks gestation in the sample of 123 infants born of HIV-infected but non-transmitting mothers (120 mothers; 3 twin pregnancies) and 46 infants born of 46 HIV-uninfected mothers. Multivariate logistic regression analysis showed a non-significant association (*p* = 0.269) of more deliveries before 37 weeks of pregnancy among mothers with CMV-DNAemia ≥ 50 copies/mL (Additional file 2: Table 6).

When we limited the multivariate analysis to infants born of HIV-infected but non-transmitting mothers, this trend showed a borderline statistical significance (OR 2.71, CI 0.94–7.88, *p* = 0.063, Table 4). This association was robust to parameter elimination (OR 2.37, CI 0.88–6.29, *p* = 0.083, Table 4). In the same analysis, time since the HIV diagnosis was a significant predictor for delivery before 37 weeks (OR 0.73, CI 0.55–0.91, *p* = 0.011, Table 4), independent of maternal age.

**Table 4:** Predictors for pre-term birth (< 37 weeks of pregnancy)

	Dependent variable: preterm birth (< 37 weeks)	
	Without variable elimination	With variable elimination
*Logistic regression results—odds ratios for preterm birth risk in HIV-infected women*		
Maternal CMV-DNA of > 50 copies/mL (yes/no)	2.71 (CI 0.94–7.88) *	2.37 (CI 0.88–6.29) *
Maternal HIV-RNA load (per 1000 copies/mL)	0.97 (CI 0.88–1.05)	
Years with HIV diagnosis	0.73 (CI 0.54–0.92) **	0.73 (CI 0.55–0.91) **
On cART (yes/no)	0.53 (CI 0.18–1.52)	
Maternal age (years)	1.06 (CI 0.98–1.15)	

Logistic regression showing the association of different variables with preterm birth in HIV-infected but non-transmitting mothers. Results are presented as odds ratios; p-values are indicated as follows: *p < 0.1, **p < 0.05, ***p < 0.01. 121 participants were analysed (excluded: 2 due to missing data, 4 stillbirths and 11 MTCT cases)

CI: 95% confidence interval, CMV: cytomegalovirus, cART: combination anti-retroviral treatment, HIV: human immunodeficiency virus

Among HIV-infected pregnant women, delivery before 37 weeks gestation was frequent (34/134, 25.4%) with lower rates observed among those with successful cART with HIV-1-RNA load of < 20 copies per mL (7/39, 17.5%). We limited the logistic regression analysis to HIV-infected but non-transmitting mothers under successful cART (≤ 20 copies/ mL). In this collective, no association of more frequent deliveries at < 37 weeks with CMV-DNAemia ≥ 50 copies/mL remained (*p* = 0.861, Additional file 2: Table 7).

### Association of antenatal CMV DNA levels with infant birth weight

An association of lower birth weight of infants born from HIV-infected mothers and/or mothers with CMV-DNAemia was noted but failed to reach statistical significance (*p* = 0.450). Further, antenatal CMV-DNAemia was associated with lower birth length in some but not all analyses (Table 1, Additional file 2: Table 1).

## Discussion

In this pilot study, we used data from UZBCS to test the association between antenatal plasma CMV-DNAemia and HIV-MTCT in a case–control design of 131 HIV-infected pregnant women and their 134 HIV exposed infants (including 3 sets of twins). Our results indicate that pregnant women ≥ 20 weeks gestational age with CMV-DNAemia > 50 copies per mL had an odds ratio of approximately 4 for HIV-1 vertical transmission within the first 6 months of life.

In our pilot study, CMV-DNAemia did not differ regarding HIV status and cART use, suggesting CMV reactivation as a potential problem in pregnant women regardless of HIV status. These results contrast with previous findings where CMV reactivation occurred more frequently in HIV-infected but non-pregnant American women, even among those on cART [22]. Furthermore, no significant association between maternal age and CMV-DNA load was apparent in our pilot study, most likely due to the much younger age of pregnant women of our cohort, in contrast to a Brazilian study that observed higher CMV-DNA loads in relatively older  women [23]. Generally, differences may potentially be related to distinct environmental exposures including presence of other co-infections in SSA compared to Western countries.

Antenatal plasma CMV-DNAemia of > 50 copies per mL was a significant predictor for HIV vertical transmission (OR 4.02, CI 1.09–15.30, *p* = 0.035), which is in agreement with previous findings [24]. Similarly, in a clinical trial of South African and American cART naïve pregnant women, urinary CMV levels were significant risk factors for CMV and HIV transmission to infants [25].

Thirty six (36) % of the HIV vertical transmission occurred early, either in utero or intrapartum among cART naïve or mothers on cART for < 30 days. Some previous studies showed much higher rates of intrauterine HIV infection of about 70% (26, 27) versus just 9% observed in our study. The difference could be that these previous studies assessed HIV vertical transmission in infants with an already confirmed CMV infection.

CMV has been shown to enhance placenta susceptibility and replication of HIV-1, and may facilitate in utero HIV transmission [28]. In line with these findings, the presence of CMV DNAemia in HIV-infected mothers might be the underlying reason behind our earlier observation of pregnant women who transmitted HIV to their infants despite having low antenatal HIV-1-RNA-loads of < 20 copies/mL [29].

We also observed higher CMV-DNA levels in pregnant women with baseline absolute CD4 counts < 200 cells/µl, in line with other reports of CMV-DNA load as a marker for immunosuppression and elevated HIV-1-RNA load [30].

In a North American study, the risk of infant hearing deficit increased with higher  antenatal plasma CMV-DNA of ≥ 17,000 copies/mL [31]. However, there is still insufficient local/African data to define reliable viral load cut-offs to indicate disease severity or justify the need for CMV-specific antiviral treatment. Our data indicate that screening for CMV-DNA in pregnant women in SSA may provide useful information regarding infant prognosis.

Secondary analyses indicated higher rates of deliveries before 37 weeks gestation in HIV-infected CMV viremic women, although without statistical significance probably due to small sample size (*p* = 0.063 in the complete model, *p* = 0.083 after variable elimination, Table 4). This trend is not surprising since CMV infection has been shown to interfere with placental development, critical for the maintenance of a healthy pregnancy [32]. Preterm deliveries may occur due to CMV-induced maternal immune activation and systemic inflammatory caused by CMV reactivation in pregnancy [9].

We observed an association with lower birth weight (> 2500 g) which also failed to reach statistical significance, probably due to the limited power of our pilot study. In other studies, maternal CMV reactivation affected placental function, leading to intra-uterine growth retardation [32], which might lead to lower infant length for age and head circumference later in life.

Our pilot study has several strengths and limitations. Extensive clinical characterization of HIV-infected pregnant women with different duration of exposures to cART compared to their HIV-uninfected counterparts is a unique strength of our study. Further, all research participants reside in highly similar environmental conditions in high-density areas of Harare resulting in an unusually homogenous study population. Limitations include the modest number of participants for which CMV-DNA levels were available and our pilot study may be underpowered for other potential adverse infant outcomes. Maternal HIV-RNA-load, the most significant factor determining HIV vertical transmission was assessed once at enrolment and no additional measurements of maternal HIV -1-RNA load are available until at exit. cCMV infections and trends are yet to be assessed in all the infants born of 600 HIV-infected and 600-uninfected pregnant women of the UZBCS, to investigate whether early HIV infection predisposes infants to CMV infection or the other way round. Further, our non-interventional study design does not allow us to distinguish whether CMV is an independent risk factor for HIV-1-MTCT or directly responsible for adverse outcomes, so further mechanistic studies are needed in this regard.

## Conclusion

Antenatal plasma CMV-DNA loads > 50 copies/mL may be an independent risk factor for HIV-1-MTCT, raising the possibility that controlling maternal CMV-DNAemia levels might improve infant health outcomes. These findings should encourage fresh attempts to implement CMV diagnostics in clinical care for pregnant women and their infants in SSA. Further studies are warranted to determine whether anti-CMV treatment may improve pregnancy outcomes and infant health in low resource settings.

## Supplementary information

**Additional file 1. Figure 1: **Scatterplot of Maternal baseline HIV-1-RNA load in copies/mL versus CMV-DNA copies/mL in log scale.

**Additional file 2.** **Table 1**: HIV-infected non-transmitting participants. Comparison of characteristics of HIV-infected non-transmitting participants between the study sample and the control sample. Data are expressed as n (%) or median (IQR); min-max unless stated otherwise. P-values to compare patient groups were calculated using the Mann-Whitney U test or Fisher’s exact test where appropriate. Missing data are indicated in the following manner: number of * = number of missing data points. Abbreviations: BMI: body mass index, CMV: cytomegalovirus, HIV: human immunodeficiency virus, WHO: world health organisation, N/A: not applicable. **Table 2**: HIV-uninfected participants. Comparison of characteristics of HIV-uninfected mothers between the study sample and the control sample. Data are expressed as n (%) or median (IQR); min-max unless stated otherwise. P-values to compare groups were calculated using Mann-Whitney U test or Fisher’s exact test where appropriate. Missing data are indicated in the following manner: number of * = number of missing data points. Abbreviations: BMI: body mass index, CMV: cytomegalovirus, HIV: human immunodeficiency virus, WHO: world health organisation, N/A: not applicable. **Table 3**: HIV-infected and uninfected study participants. Sample characteristics stratified for HIV status and CMV-DNAemia (4). Data are expressed as n (%) or median (IQR); min-max unless stated otherwise. P-values to compare patient groups were calculated using the Kruskal Wallis test, Mann-Whitney U test or Fisher’s exact test where appropriate. Missing data are indicated in the following manner: number of * = number of missing data points. Abbreviations: BMI: body mass index, CMV: cytomegalovirus, HIV: human immunodeficiency virus, WHO: world health organisation, N/A: not applicable. **Table 4**: CMV viremic and aviremic participants. Sample characteristics stratified for HIV status, cART exposure and CMV status and analysis of differences in participant groups. Data are expressed as n (%) or median (IQR); min-max unless stated otherwise. For calculation of vertical transmissions only infants that survived the first 6 weeks were included; exclusions due to death are marked with one “†” for each deceased infant. P-values to compare patient groups were calculated using the Kruskal Wallis test, Mann-Whitney U test or Fisher’s exact test where appropriate. Missing data are indicated in the following manner: number of * = number of missing data points. Abbreviations: BMI: body mass index, CMV: cytomegalovirus, cART: combination antiretroviral treatment, HIV: human immunodeficiency virus, WHO: world health organisation, N/A: not applicable. **Table 5**: Predictors of maternal CMV-DNA load. Linear regression showing the association of different variables with maternal CMV-DNA load in HIV-infected non-transmitting mothers after the removal of 0 outliers by the Tukey's fences method (Q1 – 3×IQR and Q3 + 3×IQR). Results are presented as linear coefficients; p-values are indicated as follows: *: p<0.1, **: p<0.05, ***: p<0.01. 121 participants were analysed. Abbreviations: CI: 95% confidence interval, CMV: cytomegalovirus, cART: combination antiretroviral treatment, HIV: human immunodeficiency virus, IQR: interquartile range, Q: quartile. **Table 6**: Predictors for pre-term birth (<37 weeks of pregnancy). Logistic regression showing the association of different variables with preterm birth in HIV-infected but non-transmitting mothers. Results are presented as odds ratios; p-values are indicated as follows: *: p<0.1, **: p<0.05, ***: p<0.01. 169 participants were analysed. Abbreviations: CI: 95% confidence interval, CMV: cytomegalovirus. **Table 7**: Predictors for pre-term birth (<37 weeks of pregnancy). Logistic regression showing the association of different variables with preterm birth in HIV-infected but non-transmitting mothers. Results are presented as odds ratios; p-values are indicated as follows: *: p<0.1, **: p<0.05, ***: p<0.01. 36 participants were analysed. Abbreviations: CI: 95% confidence interval, CMV: cytomegalovirus, cART: combination antiretroviral treatment, HIV: human immunodeficiency virus.

## Data Availability

The datasets obtained during this study will be available upon request to the corresponding author. Results will be published in journals with scientific quality assurance, targeting open access journals whenever possible for a wider accessibility.

## Abbreviations

BMI: Body mass index
CD4: Cluster of differentiation 4
CI: Confidence interval
CMV: Cytomegalovirus
DNA: Deoxy-ribonucleic acid
cART: Combination antiretroviral therapy
HIV: Human immunodeficiency virus
IQR: Interquartile range
MTCT: Mother to child transmission
MUAC: Mid upper arm circumference
n.s: Not significant
RNA: Ribonucleic acid
RT-PCR: Real-time polymerase chain reaction
UZBCS: University of Zimbabwe Birth Cohort Study
