# Supplementary figures and images for: Role of antenatal plasma cytomegalovirus DNA levels on pregnancy outcome and HIV-1 vertical transmission among mothers in the University of Zimbabwe birth cohort study (UZBCS)
Source: Virol J. 2021 Jan 29;18:30. doi: 10.1186/s12985-021-01494-3 (PMC7846993; doi:10.1186/s12985-021-01494-3)

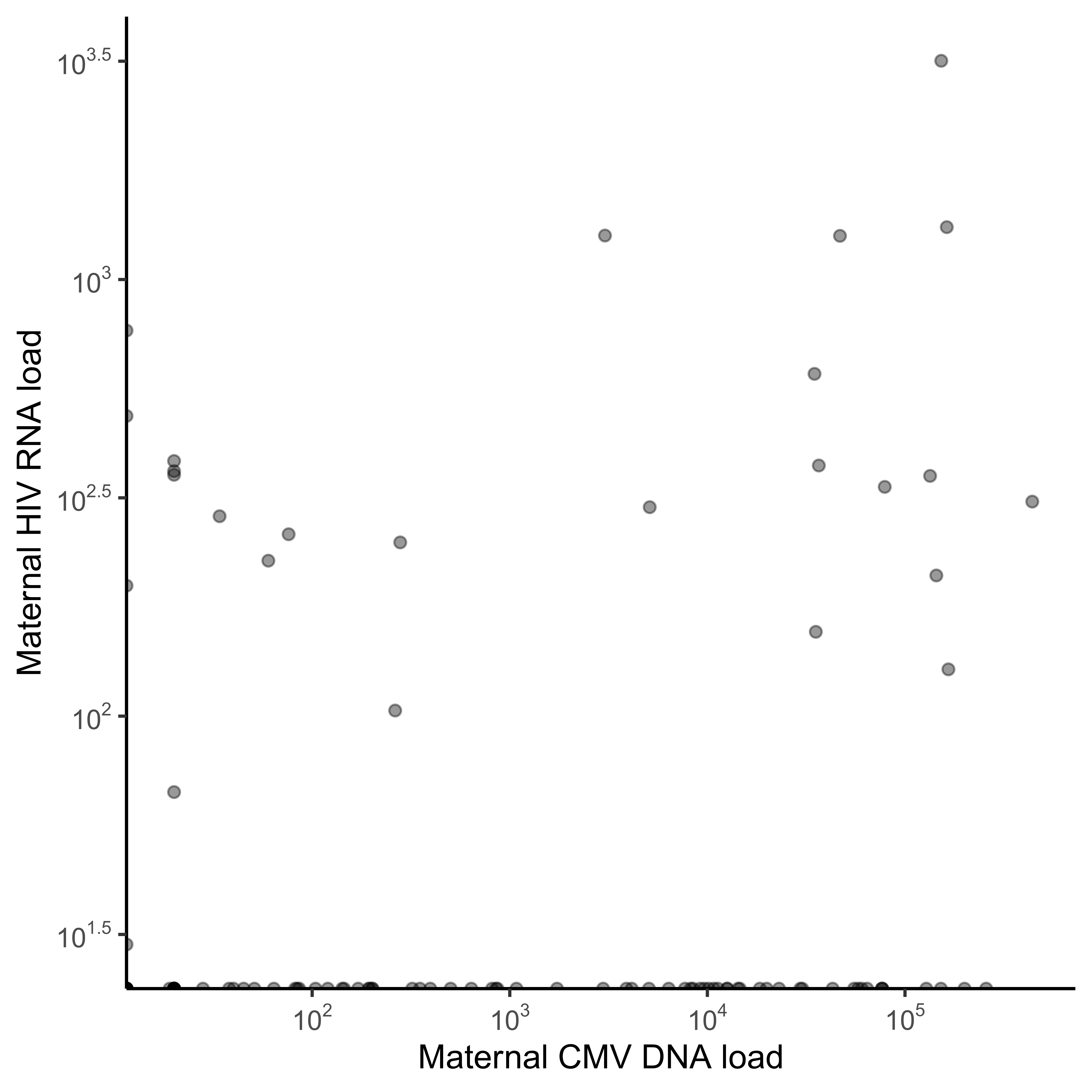

Supplement: Supplementary file 1 — Additional file 1. Figure 1: Scatterplot of Maternal baseline HIV-1-RNA load in copies/mL versus CMV-DNA copies/mL in log scale. [file 12985_2021_1494_MOESM1_ESM.tif]
