# Supplementary material for: Role of antenatal plasma cytomegalovirus DNA levels on pregnancy outcome and HIV-1 vertical transmission among mothers in the University of Zimbabwe birth cohort study (UZBCS)
Source: Virol J. 2021 Jan 29;18:30. doi: 10.1186/s12985-021-01494-3 (PMC7846993; doi:10.1186/s12985-021-01494-3)
Supplement: Supplementary file 2 — Additional file 2. Table 1: HIV-infected non-transmitting participants. Comparison of characteristics of HIV-infected non-transmitting participants between the study sample and the control sample. Data are expressed as n (%) or median (IQR); min-max unless stated otherwise. P-values to compare patient groups were calculated using the Mann-Whitney U test or Fisher’s exact test where appropriate. Missing data are indicated in the following manner: number of * = number of missing data points. Abbreviations: BMI: body mass index, CMV: cytomegalovirus, HIV: human immunodeficiency virus, WHO: world health organisation, N/A: not applicable. Table 2: HIV-uninfected participants. Comparison of characteristics of HIV-uninfected mothers between the study sample and the control sample. Data are expressed as n (%) or median (IQR); min-max unless stated otherwise. P-values to compare groups were calculated using Mann-Whitney U test or Fisher’s exact test where appropriate. Missing data are indicated in the following manner: number of * = number of missing data points. Abbreviations: BMI: body mass index, CMV: cytomegalovirus, HIV: human immunodeficiency virus, WHO: world health organisation, N/A: not applicable. Table 3: HIV-infected and uninfected study participants. Sample characteristics stratified for HIV status and CMV-DNAemia (4). Data are expressed as n (%) or median (IQR); min-max unless stated otherwise. P-values to compare patient groups were calculated using the Kruskal Wallis test, Mann-Whitney U test or Fisher’s exact test where appropriate. Missing data are indicated in the following manner: number of * = number of missing data points. Abbreviations: BMI: body mass index, CMV: cytomegalovirus, HIV: human immunodeficiency virus, WHO: world health organisation, N/A: not applicable. Table 4: CMV viremic and aviremic participants. Sample characteristics stratified for HIV status, cART exposure and CMV status and analysis of differences in participant groups. Data are ex [file 12985_2021_1494_MOESM2_ESM.docx]

# Supplementary Tables

| **Variables**  **Median (1^st^ quartile-3^rd^ quartile); min-max** | | **HIV-infected non-transmitters, N=169** | | **P-value** |
| --- | --- | --- | --- | --- |
|  | | **Randomly selected pregnant women** | **Cohort controls (not part of this pilot study)** |  |
| **Maternal outcomes (N mothers)** | | **N=120** | **N=149** |  |
| **CMV-DNA (copies/mL)** | | 0(0-0); 0- 3169 | N/A |  |
| **Maternal age (years)** | | 29 (24.8-32.8); 18.3-43.7 | 30.7 (26.9-34.4); 17.5-45.6 | 0.12 |
| **Family income per month (US dollars)** | | 231(164-349); 0-1000 | 239.5 (150-321); 0-2000 | 0.68 |
| **Size of household** | | 3 (2-4); 2-17 | 4(3-5); 1-11 | 0.19 |
| **Number of under 5s in the household** | | 1 (0-1); 0-3 | 0 (0-1); 0-4 | 0.59 |
| **Parity** | | 1 (0-2); 0-4 | 2 (1-3); 0-5 | **0.01** |
| **Hemoglobin (g/dL)** | | 11 (9.8-11.7); 3.6-14 | 10.9 (9.9-11.8); 4.6-15.5 | 0.86 |
| **Mid upper arm circumference (cm)** | | 28 (26-30); 21-39 | 28 (26-31); 20-41 | 0.14 |
| **BMI** | | 25.7 (22.8-28.2); 17.3-41.3 | 25.9 (23.8-28.2); 18.6-67.6 * | 0.22 |
| **Pregnancy outcomes (including 3 sets of twins)** | | **N=123** | **N=152** |  |
| **Delivery < 37 weeks gestational age** | **Yes** | 32 (26%) | 32 (21%) | 0.39 |
|  | **No** | 91 (74 %) | 120 (79%) |  |
| **Birth weight (g)** | | 2980(2600-3300); 1000-4130 | 3075 (2700-3345); 1547-4300 ** | 0.14 |
| **Birth weight z score (WHO Growth Reference 2016)** | | -0.8 (-1.7-(-)0.1); -6.8-1.5 | -0.6 (-1.4-(-)0); -4.6-1.8 ** | 0.14 |
| **Birth head circumference (cm)** | | 34 (34-35); 23-41***** | 34 (33-35); 28-45** | 0.94 |
| **Birth length (cm)** | | 49 (48-51); 27-56**** | 49.4 (48-51); 40-56 ** | 0.50 |
| **Birth length z score (WHO Growth Reference 2016)** | | -0.1 (-1.0-0.6); -12.1-3.7 **** | -0.1 (-1.0-0.6); -5.2-3.7 ** | 0.40 |
| **Apgar score** | | 9 (8-10); 0-10 * | 9 (9-10); 0-10 | 0.92 |

**Supplementary Table 1: HIV-infected non-transmitting participants.** Comparison of characteristics of HIV-infected non-transmitting participants between the study sample and the control sample. Data are expressed as n (%) or median (IQR); min-max unless stated otherwise. P-values to compare patient groups were calculated using the Mann-Whitney U test or Fisher’s exact test where appropriate. Missing data are indicated in the following manner: number of * = number of missing data points. **Abbreviations:** BMI: body mass index, CMV: cytomegalovirus, HIV: human immunodeficiency virus, WHO: world health organization, N/A: not applicable.

| **Variables**  **Median (1^st^ quartile-3^rd^ quartile); min-max** | | **HIV-uninfected; N=247** | | **P-value** |
| --- | --- | --- | --- | --- |
|  | | **Randomly selected mothers** | **Cohort controls (not part of this pilot study)** |  |
| **Maternal outcomes (N =247)** | | **N=46** | **N=201** |  |
| **CMV-DNA (copies/mL)** | | 0(0-0); 0-30279 | N/A |  |
| **Maternal age (years)** | | 25.6 (21.8-32); 17.6-41.7 | 26.1 (21.4-32.1); 16.7-43.1 | 0.75 |
| **Family income per month (US dollars)** | | 200 (175-300); 35-1000 | 250 (140-350); 0-1100 | 0.54 |
| **Size of household** | | 4 (3-5); 2-6 | 3 (3-5); 1-10 | 0.99 |
| **Number of under 5s in the household** | | 0 (0-1); 0-1 | 1(0-1); 0-3 | 0.28 |
| **Parity** | | 1 (0-2); 0-5 | 1 (0-2); 0-8 | 0.70 |
| **Gravida** | | 2. (2-3.8); 1-6. | 2. (1.-3.); 0-7. | 0.37 |
| **Hemoglobin (g/dL)** | | 11.5 (10.2-12.); 6.5-14 | 11.5 (10.8-12.3); 7.1-14.1 | 0.30 |
| **Mid upper arm circumference (cm)** | | 27.5 (26-30); 20-42 | 28.5 (26-31); 20-43. | 0.24 |
| **BMI** | | 25.8 (24-28.6); 19.8-39.3 * | 26.5 (23.5-30); 18.4-45.8 * | 0.39 |
| **Pregnancy outcomes** | | **N=46** | **N=201** |  |
| **Delivery < 37 weeks gestational age** | **Yes** | 8 (17.4%) | 28 (13.9%) | 0.47 |
|  | **No** | 38 (82.6%) | 173 (86.1%) |  |
| **Birth weight (g)** | | 3000 (2805-3300); 1800-3900 | 3100 (2800-3400); 900-4600 | 0.29 |
| **Birth weight z score (WHO Growth Reference 2016)** | | -0.7 (-1.2-(-)0.1); -3.8-1.1 | -0.5 (-1.2-(-)0.1); -7.2-2.3 | 0.29 |
| **Birth length (cm)** | | 49.5 (48.-52); 42-58 | 50.(49-52); 31-57* | 0.41 |
| **Birth length z score (WHO Growth Reference 2016)** | | -0 (-0.6-1.1); -4.2-4.8 | 0.1 (-0.5-1.1); -10-3.8 * | 0.50 |
| **Apgar score** | | 9 (9-10); 0-10 | 9 (9-10); 0-10 | 0.56 |

**Supplementary Table 2: HIV-uninfected participants.** Comparison of characteristics of HIV-uninfected mothers between the study sample and the control sample. Data are expressed as n (%) or median (IQR); min-max unless stated otherwise. P-values to compare groups were calculated using Mann-Whitney U test or Fisher’s exact test where appropriate. Missing data are indicated in the following manner: number of * = number of missing data points. **Abbreviations:** BMI: body mass index, CMV: cytomegalovirus, HIV: human immunodeficiency virus, WHO: world health organization, N/A: not applicable.

| **Variables**  **Median (1^st^ quartile-3^rd^ quartile); min-max** | **Stratifications; HIV status and CMV-DNAemia** | | | | **P value** |
| --- | --- | --- | --- | --- | --- |
| **HIV status (N mothers)** | **HIV-uninfected, N=46** | | **HIV-infected including vertical transmitters, N=131** | |  |
| **CMV-DNAemia (N mothers)** | **CMV aviremic, N=36** | **CMV viremic,**  **N=10** | **CMV aviremic, N=99** | **CMV viremic,**  **N=32** |  |
| **Family income per month (US dollars)** | 200 (166-293); 35-1000 | 208 (190-338); 72-520 | 230 (156-350); 0.-700 | 250 (190-340); 0-1000 | 0.87 |
| **Size of household** | 4.(2.8-5); 2.-6 | 3.5 (3-4); 2-5 | 4 (2-5); 2.-17 | 3 (3-4); 2-11 | 0.77 |
| **Number of under 5s in the household** | 0 (0-1); 0-1 | 0.5 (0.0-1.0); 0-1 | 1 (0-1); 0-3.0 | 0.(0-1); 0-3 | 0.65 |
| **Parity** | 1 (0-2); 0-4 | 1 (0.3-3.); 0-5 | 2 (1-2); 0-5 | 1 (0-2); 0-4 | 0.27 |
| **Gravida** | 2(1.8-3); 1-6 | 2.5 (2-4.8); 1-6 | 3 (2-4); 1-6 | 2.0 (1-3); 1-6 | 0.41 |
| **Hemoglobin (g/dL)** | 11.5 (10-11.9); 6.5-14.0 | 11.4 (10.7-12); 8.8-13.6 | 11 (10-11.9); 3.6-14 | 10.4 (9.4-11.3); 6.3-12.3 | **0.02** |
| **BMI** | 25.9 (24.1-29); 19.8-39.3 * | 25.0 (21.3-27.1); 20.2-31.2 | 25.9 (23-28.8);17-41 | 24.4 (22.4-28.1); 18.3-33.3 | 0.30 |
| **Pregnancy outcomes (N=180 infants, including 3 sets of twins)** | **CMV aviremic, N=36** | **CMV viremic,**  **N=10** | **CMV aviremic, N=101** | **CMV viremic,**  **N=33** |  |
| **Birth head circumference (cm)** | 34(34-35); 31-36 | 34 (33-35); 32-36 | 34 (34-35); 23-41**** | 34 (34-35); 25-38 * | 0.69 |
| **Apgar score** | 9 (9-10); 0-10 | 9 (8.3-9.8); 6-10 | 9 (8-10); 0-10 | 9 (8-9); 6-10 * | 0.19 |

**Supplementary Table 3: HIV-infected and uninfected study participants.** Sample characteristics stratified for HIV status and CMV-DNAemia (4). Data are expressed as n (%) or median (IQR); min-max unless stated otherwise. P-values to compare patient groups were calculated using the Kruskal Wallis test, Mann-Whitney U test or Fisher’s exact test where appropriate. Missing data are indicated in the following manner: number of * = number of missing data points. Abbreviations: BMI: body mass index, CMV: cytomegalovirus, HIV: human immunodeficiency virus, WHO: world health organization, N/A: not applicable.

| **Variables**  **Median (1^st^ quartile-3^rd^ quartile); min-max** | | **Stratifications, HIV status, CMV-DNAemia and duration of cART Exposures** | | | | | |  |
| --- | --- | --- | --- | --- | --- | --- | --- | --- |
| **HIV status** | | **HIV-uninfected, N=46** | | **HIV-infected including vertical transmitters, N=131** | | | |  |
| **cART exposure** | | **N/A** | | **cART naïve, N=28** | | **cART use, N=103** | |  |
| **CMV-DNAemia** | | **CMV aviremic N=36** | **CMV viremic N=10** | **CMV aviremic, N=21** | **CMV viremic, N=7** | **CMV aviremic, N= 78** | **CMV viremic,N=25** | **P-value** |
| **CMV viremic (detectable)** | **Yes** | 10 (21.7%) | | 7 (25.0%) | | 25 (24.3%) | | 0.932 |
|  | **No** | 36 (78.3%) | | 21 (75.0%) | | 78 (75.7%) | |  |
| **CMV-DNA (copies/mL)** | | N/A | 178 (125.8-344); 82-30279 | N/A | 301 (177.5-899.5); 128-1318 | N/A | 355 (227-388); 30-3169 | 0.530 |
| **Maternal age (years)** | | 24.4 (21.7-29.8); 18.1-41.7 | 26.3 (22.5-34.1); 17.6-40.1 | 26.4 (22.7-29.6); 19.2-34.5 | 25.1 (23.4-29.9); 18.3-30.7 | 30.8 (26.5-35.4); 18.4-43.7 | 28.3 (23.5-33.3); 20.9-41.7 | **0.003** |
| **Hemoglobin (g/dL)** | | 11.5 (10.0-11.9); 6.5-14.0 | 11.4 (10.7-12.0); 8.8-13.6 | 10.9 (9.7-11.4); 3.6-13.7 | 9.4 (8.6-10.4); 7.5-12.3 | 11.1 (10-11.9); 7.5-14 | 10.5 (9.4-11.2); 6.3-12.3 | **0.045** |
| **HIV-RNA of > 20 copies/mL (viremic)** | **Yes** | N/A | N/A | 19 (90.5%) | 6 (85.7%) | 45 (59.2%) ** | 17 (68%) | **0.030** |
|  | **No** | N/A | N/A | 2 (9.5%) | 1 (14.3%) | 31 (40.8%) ** | 8 (32%) |  |
| **HIV-RNA (copies per mL)** | | N/A | N/A | 12593 (4141-60364); 0-257539 | 46880 (20245-164327); 0-614706 | 83 (0-8269); 0-152000 ** | 263 (20-38140); 0-440619 | **<0.000** |
| **CD4 count cells/ul** | | N/A | N/A | 379 (292-559); 66-828 | 209.5 (112-402); 50-621 | 409 (276-511); 55-966 * | 367 (122-492); 26-1153 | 0.268 |
| **Days since HIV diagnosis** | | N/A | N/A | 65 (28-82); 12-156 | 30 (20.5-36); 0-64 | 395 (94.5-1687.8); 9-7597 | 875 (116-2147); 24-8054 | **<0.000** |
| **Days of cART use at enrolment** | | N/A | N/A | N/A | N/A | 322 (27-1512); 1-3942 | 575 (43-1240); 1-4327 | **<0.000** |
| **Pregnancy outcomes, (including 3 sets of twins)** | | **CMV aviremic N=36** | **CMV viremic N=10** | **CMV aviremic, N=23** | **CMV viremic, N=7** | **CMV aviremic, N= 79** | **CMV viremic**  **N=25** |  |
| **Delivery < 37 weeks gestational age** | **Yes** | 7 (19.4%) | 1 (10%) | 7 (31.8%) | 5 (62.5%) | 15 (19%) | 7 (28%) | 0.093 |
|  | **No** | 29 (80.6%) | 9 (90%) | 15 (68.2%) | 3 (37.5%) | 64 (81%) | 18 (72%) |  |
| **Birth weight (g)** | | 3075 (2737.5-3325); 1800-3885 | 3000 (2925-3038); 2600-3900 | 3000 (2757.5-3213); 2300-3900 | 2550 (1750-3181); 1000-3500 | 2900 (2600-3350); 1300-4130 | 2800 (2600-3300); 2000-3600 | 0.589 |
| **Birth weight z score (WHO Growth Reference 2016)** | | -0.6 (-1.3-(-)0.0); -3.8-1.1 | -0.7 (-0.9-(-)0.7); -1.7-1.1 | -0.7 (-1.3-(-)0.3); -2.4-1.1 | -1.8 (-4.1-(-)0.4); -6.8-0.3 | -1.0 (-1.7-0.0); -5.5-1.5 | -1.2 (1.7(-) 0.1); -3.2-0.5 | 0.589 |
| **Birth head circumference (cm)** | | 34 (34-35); 31-36 | 34 (33.3-35); 32-36 | 34.5 (34-35.3); 23-38 ** | 34 (33.5-35); 25-38 * | 34 (33-35); 23-41** | 34 (34-35); 31-36 | 0.778 |
| **Birth length (cm)** | | 49 (48-51); 42-55 | 51 (49.3-52); 47- 56 | 48 (47-50.3); 45-54 ** | 48.0 (40-49); 27-56 * | 49 (48-51); 36-55* | 49 (48-50);40-56 | 0.092 |
| **Birth length z score (WHO Growth Reference 2016)** | | -0.1 (-0.7-0.6); -4.2-4.8 | 0.8 (-0.0-1.5); -1.5-3.7 | -1.0 (-1.3-0.5); -2.6-2.6 ** | -1.0 (-5.1-(-)0.3); -12.1-3.7 * | -0.1 (-0.9-0.6); -7.3-2.7 * | -0.1 (-0.6-0.5); -4.9-3.2 | 0.083 |
| **Vertical transmission by 6 months of age** | **Yes** | N/A | N/A | 0 (0%) † | 1 (14.3%) | 5 (6.6%) ††† | 5 (20%) | 0.082 |
|  | **No** | N/A | N/A | 21 (100%) † | 7 (85.7%) | 71 (93.4%) ††† | 20(80%) |  |

**Supplementary Table 4: CMV viremic and aviremic participants.** Sample characteristics stratified for HIV status, cART exposure and CMV status and analysis of differences in participant groups. Data are expressed as n (%) or median (IQR); min-max unless stated otherwise. For calculation of vertical transmissions only infants that survived the first 6 weeks were included; exclusions due to death are marked with one “†” for each deceased infant. P-values to compare patient groups were calculated using the Kruskal Wallis test, Mann-Whitney U test or Fisher’s exact test where appropriate. Missing data are indicated in the following manner: number of * = number of missing data points. **Abbreviations:** BMI: body mass index, CMV: cytomegalovirus, cART: combination anti-retroviral treatment, HIV: human immunodeficiency virus, WHO: world health organization, N/A: not applicable.

| **Linear regression results - linear coefficients for maternal CMV viral load** | | |
| --- | --- | --- |
|  | Dependent variable: maternal CMV viral load | |
|  | Without variable elimination | With variable elimination |
| **Maternal HIV-RNA of > 20 copies/mL (yes/no)** | 23.15 (CI: -82.16-129.62) | 42.63 (CI: -56.75-142.00) |
| **On cART (yes/no)** | -73.77 (CI: -188.79-41.26) |  |
| **Maternal age (years)** | 1.33 (CI: -7.34-10.01) |  |
| **Maternal BMI** | -1.02 (CI: -11.81-9.78) |  |
| **Number of children under five years** | -43.97 (CI: -110.59-22.66) |  |

**Supplementary Table 5: Predictors of maternal CMV-DNA load.** Linear regression showing the association of different variables with maternal CMV-DNA load in HIV-infected non-transmitting mothers after the removal of 0 outliers by the Tukey's fences method (Q1 – 3×IQR and Q3 + 3×IQR). Results are presented as linear coefficients; p-values are indicated as follows: *: p<0.1, **: p<0.05, ***: p<0.01. 121 participants were analyzed. **Abbreviations**: CI: 95% confidence interval, CMV: cytomegalovirus, cART: combination anti-retroviral treatment, HIV: human immunodeficiency virus, IQR: interquartile range, Q: quartile.

| **Logistic regression results - odds ratios for preterm birth risk** | |
| --- | --- |
|  | Dependent variable: preterm birth (< 37 weeks) |
|  | Without variable elimination |
| **Maternal CMV-DNA of > 50 copies/mL  (yes/no)** | 1.59 (CI: 0.68-3.56) |
| **Maternal age (years)** | 1.02 (CI: 0.96-1.08) |

**Supplementary Table 6: Predictors for pre-term birth (<37 weeks of pregnancy).** Logistic regression showing the association of different variables with preterm birth in HIV-infected but non-transmitting mothers. Results are presented as odds ratios; p-values are indicated as follows: *: p<0.1, **: p<0.05, ***: p<0.01. 169 participants were analysed.

**Abbreviations:** CI: 95% confidence interval, CMV: cytomegalovirus.

| **Logistic regression results - odds ratios for preterm birth risk in HIV-infected women under successful cART** | |
| --- | --- |
|  | Dependent variable: preterm birth (< 37 weeks) |
|  | Without variable elimination |
| **Maternal CMV-DNA of > 50 copies/mL  (yes/no)** | 1.24 (CI: 0.06-12.15) |
| **Years with HIV diagnosis** | 0.75 (CI: 0.47-1.06) |
| **Maternal age (years)** | 1.08 (CI: 0.90-1.30) |

**Supplementary Table 7: Predictors for pre-term birth (<37 weeks of pregnancy).** Logistic regression showing the association of different variables with preterm birth in HIV-infected but non-transmitting mothers. Results are presented as odds ratios; p-values are indicated as follows: *: p<0.1, **: p<0.05, ***: p<0.01. 36 participants were analysed.

**Abbreviations:** CI: 95% confidence interval, CMV: cytomegalovirus, cART: combination anti-retroviral treatment, HIV: human immunodeficiency virus.
